# Supplementary material for: A gut pathobiont synergizes with the microbiota to instigate inflammatory disease marked by immunoreactivity against other symbionts but not itself
Source: Sci Rep. 2017 Dec 18;7:17707. doi: 10.1038/s41598-017-18014-5 (PMC5735134; doi:10.1038/s41598-017-18014-5)
Supplement: Supplementary file 1 — Supplementary Info [file 41598_2017_18014_MOESM1_ESM.pdf]

Supplementary Information

A gut pathobiont synergizes with the microbiota to instigate inflammatory disease marked by immunoreactivity against other symbionts but not itself

João Carlos Gomes-Neto, Hatem Kittana, Sara Mantz, Rafael R. Segura Munoz, Robert J. Schmaltz, Laure B. Bindels, Jennifer Clarke, Jesse M. Hostetter, Andrew K. Benson, Jens Walter, Amanda E. Ramer-Tait

Supplementary Methods

H. bilis **qPCR assay.** The criteria used to validate this newly developed qPCR primer set for quantification of *H. bilis* was based on our previously published work [1]. Briefly, we used a combination of in silico, in vitro and in vivo approaches to determine assay sensitivity and specificity. First, we manually designed the primer set after aligning the *H. bilis* 16S rRNA with all the ASF bacterial counterparts using a Clustal W algorithm for multiple sequence alignment from the DNA Lasergene 11 Software package (DNASTAR Inc., Madison, WI) [1]. Second, we confirmed that the primers were specific to *H. bilis* via searching multiple bacterial sequence databases [1]. In vitro specificity testing was achieved using a combination of two approaches: (i) testing the *H. bilis* primer set against either *H. bilis* or individual ASF 16S rRNA cloned into plasmid vectors [1]; (ii) by generating a mock community containing all the plasmids expressing each ASF 16S rRNA gene in the presence or absence of the plasmid expressing the *H. bilis* 16S rRNA gene. All qPCR products (i.e., from individual cross-check or mock communities) were then Sanger sequenced to confirm the presence of the *H. bilis* amplicon via BLAST [1]. In

addition, primer set specificity was checked by analyzing the melting curves obtained for each reaction to identify the melting peaks corresponding to the true product based on the positive control. The degree of fluorescence for determining the cycle threshold value was set to the Mastercycler software default across all reactions (i.e., fluorescence cut-off of at least 100).

Finally, the in vivo validation was performed using samples from germ-free, ASF and Conventional C3H/HeN adult mice colonized with or without *H. bilis* as described in the Methods section. Upon DNA extraction [2], the qPCRs were performed as described in the Methods section. We also used published genus-specific *Helicobacter* primers to ascertain the free or positive status of our experimental animals for *H. bilis* [3]. Sensitivity of our assay was determined by producing plasmid (i.e., cloned with *H. bilis* 16S rRNA gene) based standard curves that were assayed in triplicate. Standard curve data were analyzed using GraphPad Prism 7, whereby a linear regression model was run to determine slope, intercept, and statistics such as the R-squared and Pearson correlation coefficient. The predicted total *H. bilis* abundance was determined using a copy number approach and took into account the total eluted DNA and cecal weight (grams) as previously described [1]. The predictive linear equation used for calculations was: $Y = -3.0659X + 33.074$, where Y = mean cycle threshold value and $X = \log_{10}$ total bacterial abundance, $R^2 = 0.985$, efficiency = 1.12. The limit of detection was determined to be 150 bacteria per sample.

Cecal explant cultures. Briefly, cecal tissues were washed in sterile 1X PBS (Corning™ Cellgro™, Thermo Fisher Scientific Inc.) to remove feces, cut into 1 cm pieces, and shaken at 280 x g in washing media for 30 min at 25°C. Washing media consisted of RPMI (RPMI-1640

medium 1X with 2.05 mM of L-Glutamine, Corning™ Cellgro™) supplemented with 2% Penicillin/Streptomycin (100X Penicillin/Streptomycin solution, Corning™ Cellgro™) and 0.5% Gentamycin (10,000 µg/mL Gentamicin Sulfate, Corning™ Cellgro™). Each piece of tissue was placed in an individual well of a 96-well flat bottom well plate (Falcon or Corning) with 200 µL of complete culture medium (same formulation as the washing media, but also included 1% L-Glutamine (200 mM L-Glutamine Solution, Corning™ Cellgro™), 0.1% of a 50 mM 2-Mercaptoethanol solution (Thermo Fisher Scientific Inc.) and 1% Pyruvate (100 mM Sodium Pyruvate Solution, Corning™ Cellgro™). Cultures were incubated at 37°C with 5% CO_2 for 24 hr. Culture supernatants were collected and stored at -20°C until assayed.

CD4^{+} T cell stimulation. One day prior to necropsying experimental mice, spleens were harvested from naïve male and female C3H/HeN Conv donor mice to prepare a single-cell suspension of feeder splenocytes as previously described [4]. Cells were enumerated and their viability checked using trypan blue reagent (Trypan Blue Solution 0.4%, Sigma® Life Science, Sigma-Aldrich, Saint Louis, MO) and a Cellometer Auto 2000 Cell Viability Counter (Nexcelom Bioscience, LLA, Lawrence, MA). The concentration of splenocyte feeder cells was adjusted to 20 million cells/1.8 mL in a 15-mL conical tube using complete tissue culture media (CTCM; Dulbecco's modified Eagle's medium containing 4.5 mg of glucose/ml, 2 mM L-glutamine, 100 U penicillin, 100 g streptomycin/ml, 25 mM HEPES, 0.05 M 2-mercaptoethanol, 10% fetal bovine serum).

Whole-cell bacterial sonicates (i.e., individual ASF and *H. bilis* antigens), prepared as previously described [5], were mixed individually with aliquots of feeder cells at a final

concentration of 200 μg/mL. Cells were incubated overnight at 37°C with 5% CO_2. Prior to incubation with purified $CD4^+$ T cells, all feeder cells were treated with 50 μg/mL filtered-sterilized mitomycin C solution (Sigma) as described [4] and incubated at 37°C for 20 min with 5% CO_2. Thereafter, feeder cells were washed 5X with an excess volume of CTCM. Feeder cell suspensions were adjusted to a final concentration of 2 million live cells/mL prior to plating 0.2 million feeder cells in 100 μL CTCM per well of a 96-well U-bottom plate with purified $CD4^+$ T cells.

To isolate purified $CD4^+$ T cells, single-cell suspensions of mesenteric lymph node (MLN) cells were prepared as previously described [6] by pooling MLN from 2 to 3 mice per treatment. $CD4^+$ T cells were isolated from MLN single-cell suspensions via magnetic depletion (negative selection) using a mouse $CD4^+$ T cell isolation kit (Miltenyi Biotec, Auburn, CA) according to the manufacturer's protocol. Cells were subjected to two passes through an AutoMACS Pro magnetic cell separator. After isolation, T cells were counted using a Cellometer 2000 (Nexcelom Bioscience, Lawrence, MA) and the concentration was adjusted to 1 million live cells/mL prior to plating 0.1 million cells in 100 μL CTCM per well of a 96-well U-bottom plate. Purified $CD4^+$ T cells and antigen-pulsed feeder cells were co-cultured for 72 hr at 37°C for 20 min with 5% CO_2. Unpulsed feeder cells plated with $CD4^+$ T cells served as the unstimulated controls. Cultures containing only unpulsed feeder cells served as background controls. Culture supernatants were harvested after 72 hrs for cytokine quantification and stored at -20°C until use.

Flow cytometry. Cells were stained with the following antibodies or the appropriate corresponding isotype controls as follows. All antibodies were obtained from eBioscience, Inc. (San Diego, CA). Assessing $CD4^+$ T cell purity after isolation: FITC anti-mouse CD4 (GK1.5, 1:500 dilution). Effector/memory (EM) $CD4^+$ T cells ($CD62L^{low}CD44^{high}$): PE-Cy7 anti-mouse CD4 (GK1.5; 1:800 dilution), APC-eFluor 780 anti-mouse CD62L (MEL-14; 1:500 dilution) and FITC anti-human/mouse CD44 (IM7; 1:500 dilution). Active B cells ($CD19^+CD23^-$): PE anti-mouse CD19 (eBio1D3; 1:800 dilution) and PE-Cy7 anti-mouse CD23 (B3B4; 1:200 dilution). Total Tregs: PE-Cy7 anti-CD4, PE anti-mouse CD25 (PC61.5; 1:250 dilution) and FITC anti-mouse Foxp3 (FJK-16s; 1:100 dilution). Differentiation between natural and inducible Tregs was achieved via the addition of PerCP-eFluor 710 anti-mouse CD304 (Neuropilin-1; 3DS304M; 1:400 dilution) and APC anti-Helios (22F6; 1:20 dilution). Stained cells were fixed using BD™ Stabilizing Fixative (BD Biosciences, San Jose, CA) according to manufacturer instructions.

Intracellular cytokine staining was performed as previously described [6]. Briefly, 0.5 million total mesenteric lymph node cells were plated in 100 μL per well of a 96-well plate and stimulated with a 1X cocktail of phorbol 12-myristate 13-acetate and ionomycin (Cell Stimulation Cocktail 500X, eBioscience Inc.) and Brefeldin A (Brefeldin A 1000X, eBioscience Inc.) for 5 hrs at 37°C with 5% CO_2. Cells were harvested and surfaced stained for CD4 and then subsequently stained intracellularly for IL-17A and IFN-γ (Intracellular Fixation & Permeabilization Reagents, eBioscience Inc.) using the following antibodies and/or their isotype controls: PerCP-eFluor 710 anti-mouse CD4 (GK1.5; 1:250 dilution), PE-Cy7 anti-mouse/rat IL-17A (eBio17B7; 1:100 dilution), eFluor 660 anti-mouse IFN-γ (XMG1.2; 1:100 dilution).

Evaluation of EM $CD4^+$ T cells secreting $IL17A^+$ and/or IFN-γ^+ included staining anti-CD62L and anti-CD44 as described above.

Cells were analyzed using a BD FACSCanto II (BD Biosciences, San Jose, CA). Data were analyzed using FlowJo v10.2 (FlowJo™, LLC, Ashland, OR). During data analysis, gates were first drawn on singlet events (FSC-H vs FSC-A) and then on lymphocytes (FSC-A vs SSC-A). Populations of interest were subsequently evaluated; gates were drawn based on fluorescence minus one controls. Representative graphs of depicting all gating strategies can be seen in Fig. S11A-E and S12A-F.

Immunoglobulin A (IgA)-based bacterial cell sorting and quantification. Sorting of IgA bound bacteria was performed using an adaptation of a previously described method[7]. Specifically, 100-400 mg of frozen cecal contents were placed in Fast Prep Lysing Matrix D tubes containing ceramic beads (MP Biomedical, Solon, OH) and incubated in 1 mL of 1X Phosphate Buffered Saline (1X PBS, Corning™ Cellgro™) for 1 hr on ice. Samples were homogenized by bead beating for 5 s (Minibeadbeater; Biospec, Bartlesville, OK), placed on ice and then centrifuged at 50 x g for 15 min at 4°C. Supernatants were removed (100 µL/sample), washed with 1 mL staining buffer containing 1% (w/v) Bovine Serum Albumin (BSA, Thermo Fisher Scientific Inc.) in 1X PBS and then centrifuged at 8,000 x g for 5 min at 4°C before resuspension in 1 mL staining buffer. A sample of this suspension (200 µL) was stored at -20°C as the pre-sorted raw material for bacterial quantification. After an additional wash, pellets were resuspended in 100 µL blocking buffer containing the staining buffer in addition to 20% Normal

Rat Serum (Jackson ImmunoResearch, West Grove, PA). Samples were then incubated for 20 min on ice.

Without washing, samples were stained with 100 µL staining buffer containing PE-conjugated anti-mouse IgA (1:12.5; eBioscience clone mA-6E1) for 30 min on ice. Samples were then washed 3 times with 1 mL staining buffer. Anti-IgA stained samples were resuspended in 1 mL of staining buffer containing 50 µL anti-PE Magnetic Activated Cell Sorting (MACS) beads (Miltenyi Biotec) and were incubated for 15 min at 4°C. Thereafter, samples were washed and centrifuged twice (10,000 x g, 5 min, 4°C) with 1 mL staining buffer, prior to resuspension in 1 mL of staining buffer for sorting MACS (Possel_s program on an AutoMACS pro; Miltenyi Biotec). After MACS separation, the entire negative fraction was centrifuged at 10,000 x g for 5 min at 4°C and resuspended in 400 µL of cold 1X PBS prior to bacterial DNA extraction and qPCR analysis. The positive fraction was then further purified via Fluorescence Activated Cell Sorting (FACS, see Fig. S10A for FACS output). For each sample, approximately 2 million IgA-positive events were collected, pelleted after centrifugation at 10,000 x g for 5 min at 4°C, following resuspension in 400 µL of cold 1X PBS prior to DNA extraction and qPCR analysis. All samples were kept at -20°C prior to bacterial quantification. The pre-sorted samples were also centrifuged at 10,000 x g for 10 min at room temperature prior to adding 400 µL of cold 1X PBS before DNA extraction and bacterial quantification.

Four hundred µL of each sample (i.e., pre-sorted, IgA+ or IgA– fractions) were placed into sterile 2 mL tubes containing 300 mg of zirconium beads (Thermo Fisher Scientific Inc.) in addition to 300 µL of the lysis buffer (200mM NaCl, 100mM Tris Base, 20 mM EDTA, 20mg/mL lysozyme)[2], 200 µL of 10 % sodium dodecyl sulfate solution and 500 µL of

Phenol:Chloroform:Isoamyl Alcohol (PCI 25:24:1) prior to incubation on ice for 4 min. Thereafter, samples were homogenized by bead beating for 2 min (Minibeadbeater; Biospec), 2 min on ice, and then another 2 min of bead beating; samples were placed on ice prior to centrifugation at 6,000 x g for 5 min at 4°C. The top layer was then transferred to a 2 mL sterile snap-cap tube (Thermo Fisher Scientific Inc.) at a ratio of 1:1 with PCI (300 µL of each) prior to homogenization. Samples were centrifuged at 16,100 x g for 3 min at room temperature and the top layer was transferred into 2 mL sterile snap-cap tubes. Two volumes of 100% ethanol (Thermo Fisher Scientific Inc.) were added prior to storing samples at -80°C for 2 hours. Samples were then centrifuged at 21,130 x g for 20 min and the supernatant discarded without disturbing the DNA pellet. Pellets were washed by adding 500 µL 70% ethanol and centrifuging at 21,130 x g for 20 min before drying the tube for 30 min at room temperature. Lastly, DNA was eluted in 200µL of Tris-EDTA Buffer (Thermo Fisher Scientific Inc.) and stored at -20°C until quantification and qPCR analysis.

ASF quantification of DNA samples from pre-sorted, IgA+ or IgA– fractions was performed as previously described[1]. *H. bilis* was quantified as described above (see ***H. bilis* qPCR assay**) using the following predictive linear equation for calculations: $Y = -2.9125X + 34.313$, where Y = mean cycle threshold value and X = $\log_{10}$ total bacterial abundance, $R^2 = 0.995$ and efficiency = 1.20, with the limit of detection determined to be 97 bacteria per sample. All qPCR reactions were run using the Maxima SYBR Green qPCR Master Mix 2X (Thermo Fisher Scientific Inc.) as previously described[1]. Prior to qPCR, DNA was quantified using the Quant-iT™ PicoGreen® dsDNA Broad Range and High Sensitivity Reagents (Thermo Fisher Scientific Inc.) as previously described[1]. For pre-sorted samples, 10 ng of DNA template (1

μL/sample) was used per reaction; 10 μL of IgA+ and IgA – fractions were used per reaction. A total reaction volume of 25 μL was used for all samples, which were tested in duplicate.

Final calculations for the relative abundance of each ASF member and *H. bilis* in all samples were made on an individual animal basis. The $\log_{10}$ total number of bacteria was initially calculated using linear equations followed by normalization to 100 ng of DNA template. Thereafter, the mean relative abundance (% of each taxon) was estimated. The ratio between the mean % of each taxon for the IgA positive and IgA negative fractions for a given animal (i.e., IgA index) was then used for statistical analysis to make comparisons between DSS treated versus control treatments. A ratio of 1 indicated no difference in relative abundance of a given bacterium between the IgA positive fraction and the corresponding IgA negative fraction.

Statistical analysis. Summary statistics were calculated for all treatments to assess the overall quality of the data set, including normality. Outliers were statistically identified using the ROUT test (Q = 1%); however, they were only removed if the value was biologically implausible given the methodology used for its measurement. An unpaired non-parametric Mann-Whitney Test was used to analyze targeted pairwise differences within the following datasets: gross and histopathological cecal scores, *H. bilis* and individual ASF abundances, proportion of EM $CD4^+$ T cells, and chemokine and cytokine concentrations. A parametric, unpaired, two-tailed T-test was used for comparisons made for cellularity of effector (IL-17A$^+$ and/or IFN-γ^+), regulatory $CD4^+$ T cells in addition to active B cells, and for comparing the IgA index across DSS treated vs non-treated groups in the assembly experiment. A non-parametric Kruskal-Wallis one-way ANOVA followed by post-hoc pairwise comparison with Dunn's test was used to compare gross

cecal scores and *H. bilis* abundance across DSS treated groups, and for individual ASF abundances in cecal contents and tissues. All those statistical analyses were performed using GraphPad Prism 7 (version 7.0a, 2016, GraphPad Software, Inc., La Jolla, CA) using the significance cut-off of $P < 0.05$.

The following statistical analyses were all conducted using R software, version 3.3.1 (R Core Team 2016, R Foundation for Statistical Computing, Vienna, Austria). To visualize the ASF community structure for individual mice, a Principal Component Analysis (PCA) of individual bacterial species abundance across different treatments was performed using scaled variables for the total number of bacteria/gram of cecal contents. This analysis was performed using the prcomp() function from the stats package. The autoplot() function from the ggplot2 package was used to generate the final plot. Scaling the variables was necessary to avoid bias due to the heterogeneity of variances across ASF member abundances that would end up inflating PC1. To compare ASF community similarities across groups and determine the factor(s) explaining variability, three analyses were performed: i) Betadisper analysis was carried out using the betadisper() function to analyze the homogeneity of the Bray-Curtis dissimilarity coefficient (BCDC) calculated using the abundances of each ASF member (total number of bacteria/gram of cecal content) between and within groups. For that analysis, the average distance to the group centroid was calculated using Euclidean's distances between BCDC for each data point in the treatment group. The between and within group variances were then analyzed via ANOVA followed by post-hoc Tukey's test. ii) The analysis of similarity (ANOSIM) used BCDC values for each experimental unit (animal) to compare differences between and within groups for the ASF abundance. This analysis generated an R statistic ranging

from -1 to 1. The closer the value to 1, the greater the dissimilarity between groups than within groups. Values below 0 suggested that dissimilarities were greater within rather than between groups, indicating that treatment was not influencing the outcome. The BCDC value was calculated using the vegdist() function. The ANOSIM was performed using the anosim() function where BCDC was the outcome of interest and treatment groups were the explanatory variables. iii) PERMANOVA analysis was performed using the adonis() function to identify the contribution of explanatory variables to the overall variability in the dataset. A variable would have a strong contribution depending on the interpretation of two values: R^2 and calculated *P*-value. All these analyses were completed using the Vegan package. The significance cut-off used across all analyses was $P < 0.05$. A Spearman's rank correlation analysis was performed to determine if there were correlations between the pathobiont and any other ASF member abundance in diseased versus non-diseased animals for both cecal contents and tissues. An arbitrary cut-off value of +0.7 was used to define a strong positive correlation between two bacterial species.

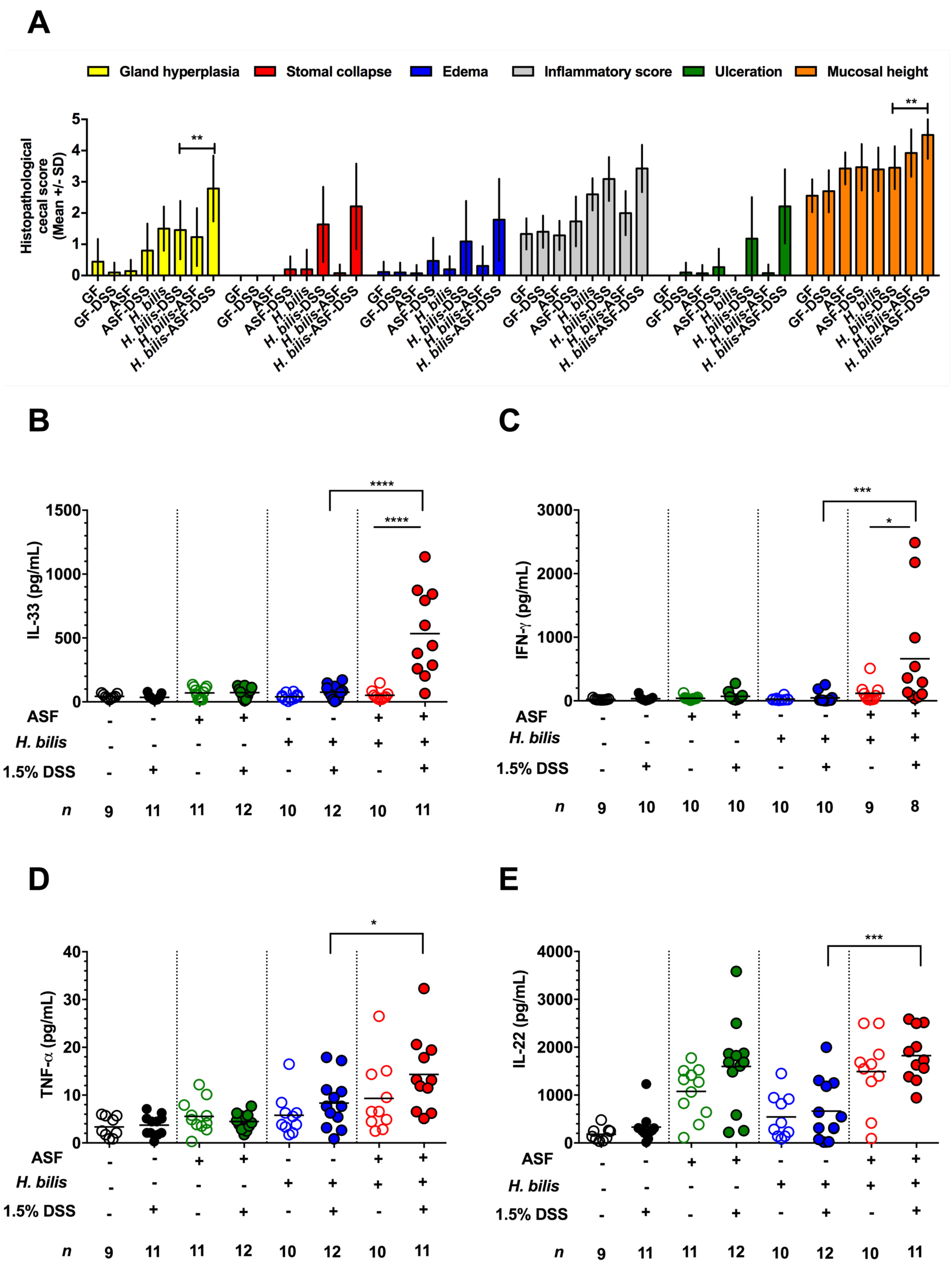
A
Gland hyperplasia
Stomal collapse
Edema
Inflammatory score
Ulceration
Mucosal height
Histopathological cecal score (Mean +/- SD)
0
1
2
3
4
5
**
**
GF
GF-DSS
ASF
ASF-DSS
H. bilis
H. bilis-DSS
H. bilis-ASF
H. bilis-ASF-DSS
B
IL-33 (pg/mL)
0
500
1000
1500
****
****
ASF
H. bilis
1.5% DSS
n
9
11
11
12
10
12
10
11
C
IFN-γ (pg/mL)
0
1000
2000
3000
***
*
ASF
H. bilis
1.5% DSS
n
9
10
10
10
10
10
9
8
D
TNF-α (pg/mL)
0
10
20
30
40
*
ASF
H. bilis
1.5% DSS
n
9
11
11
12
10
12
10
11
E
IL-22 (pg/mL)
0
1000
2000
3000
4000
***
ASF
H. bilis
1.5% DSS
n
9
11
11
12
10
12
10
11

Figure S1. The *Helicobacter* pathobiont synergized with the resident microbiota (ASF) to promote specific histopathological alterations and a pro-inflammatory response during disease. (**A**) Histopathological parameters in cecal tissues from germ-free (GF), ASF-bearing, *H. bilis* mono-associated and *H. bilis* colonized ASF-bearing mice treated with 1.5% DSS or left untreated (*n* = 9-15 animals per treatment). (**B-E**) Pro-inflammatory cytokines in cecal explants from GF, ASF-bearing, *H. bilis* mono-associated and *H. bilis* colonized ASF bearing mice treated with 1.5% DSS or left untreated (*n* = 9-12 animals per treatment). (**B-E**) Horizontal bars represent group means in all graphs. (**A-E**) Asterisks depict the degree of significance for differences as determined by a non-parametric unpaired Mann-Whitney test (i.e., cytokines) or using unpaired parametric T-test (i.e., total number of Th17 cells) with a two-tailed distribution for *P*-value calculations (* $P < 0.05$, ** $P \leq 0.01$, *** $P \leq 0.001$ and **** $P \leq 0.0001$). Only significant differences between treatments are presented in the graphs. Experiments were performed using male and female C3H/HeN mice at 8-10 wks of age; mice were colonized with *H. bilis* for 3 wks.

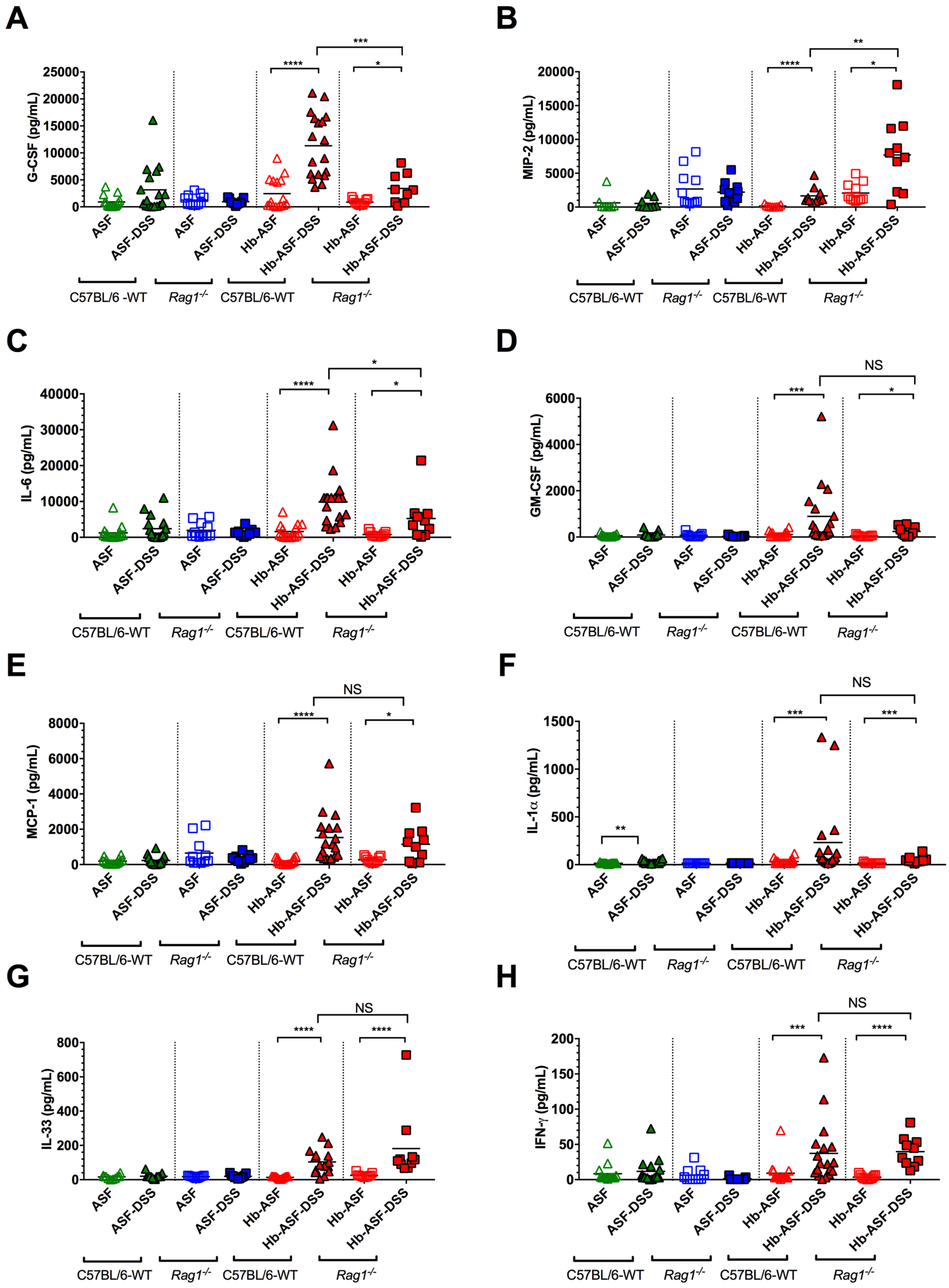
A
G-CSF (pg/mL)
25000
20000
15000
10000
5000
0
****
***
*
ASF
ASF-DSS
ASF
ASF-DSS
Hb-ASF
Hb-ASF-DSS
Hb-ASF
Hb-ASF-DSS
C57BL/6 -WT
Rag1-/-
C57BL/6-WT
Rag1-/-
B
MIP-2 (pg/mL)
20000
15000
10000
5000
0
****
**
*
ASF
ASF-DSS
ASF
ASF-DSS
Hb-ASF
Hb-ASF-DSS
Hb-ASF
Hb-ASF-DSS
C57BL/6-WT
Rag1-/-
C57BL/6-WT
Rag1-/-
C
IL-6 (pg/mL)
40000
30000
20000
10000
0
****
*
*
ASF
ASF-DSS
ASF
ASF-DSS
Hb-ASF
Hb-ASF-DSS
Hb-ASF
Hb-ASF-DSS
C57BL/6-WT
Rag1-/-
C57BL/6-WT
Rag1-/-
D
GM-CSF (pg/mL)
6000
4000
2000
0
***
NS
*
ASF
ASF-DSS
ASF
ASF-DSS
Hb-ASF
Hb-ASF-DSS
Hb-ASF
Hb-ASF-DSS
C57BL/6-WT
Rag1-/-
C57BL/6-WT
Rag1-/-
E
MCP-1 (pg/mL)
8000
6000
4000
2000
0
****
NS
*
ASF
ASF-DSS
ASF
ASF-DSS
Hb-ASF
Hb-ASF-DSS
Hb-ASF
Hb-ASF-DSS
C57BL/6-WT
Rag1-/-
C57BL/6-WT
Rag1-/-
F
IL-1α (pg/mL)
1500
1000
500
0
**
***
NS
***
ASF
ASF-DSS
ASF
ASF-DSS
Hb-ASF
Hb-ASF-DSS
Hb-ASF
Hb-ASF-DSS
C57BL/6-WT
Rag1-/-
C57BL/6-WT
Rag1-/-
G
IL-33 (pg/mL)
800
600
400
200
0
****
NS
****
ASF
ASF-DSS
ASF
ASF-DSS
Hb-ASF
Hb-ASF-DSS
Hb-ASF
Hb-ASF-DSS
C57BL/6-WT
Rag1-/-
C57BL/6-WT
Rag1-/-
H
IFN-γ (pg/mL)
200
150
100
50
0
***
NS
****
ASF
ASF-DSS
ASF
ASF-DSS
Hb-ASF
Hb-ASF-DSS
Hb-ASF
Hb-ASF-DSS
C57BL/6-WT
Rag1-/-
C57BL/6-WT
Rag1-/-

Figure S2. The absence of an adaptive immune system limited development of a pro-inflammatory response during pathobiont-induced disease. (**A-H**) Pro-inflammatory cytokines and chemokines in cecal explants from ASF-bearing C57BL/6 wild type (WT) and *Rag1*$^{-/-}$ mice harboring the ASF and colonized with or without *H. bilis* and either treated with 2% DSS or left untreated (n = 8-18 animals per treatment). Horizontal bars represent group means in all graphs. Asterisks depict the degree of significance for differences as determined by a non-parametric unpaired Mann-Whitney test using a two-tailed distribution for P-value calculations (* $P < 0.05$, ** $P \leq 0.01$, *** $P \leq 0.001$ and **** $P \leq 0.0001$ and NS = not significant $P \geq 0.05$). Experiments were performed using male and female C57BL/6 (WT or *Rag1*$^{-/-}$) mice at 8-10 wks of age; mice were colonized with *H. bilis* for 3 wks.

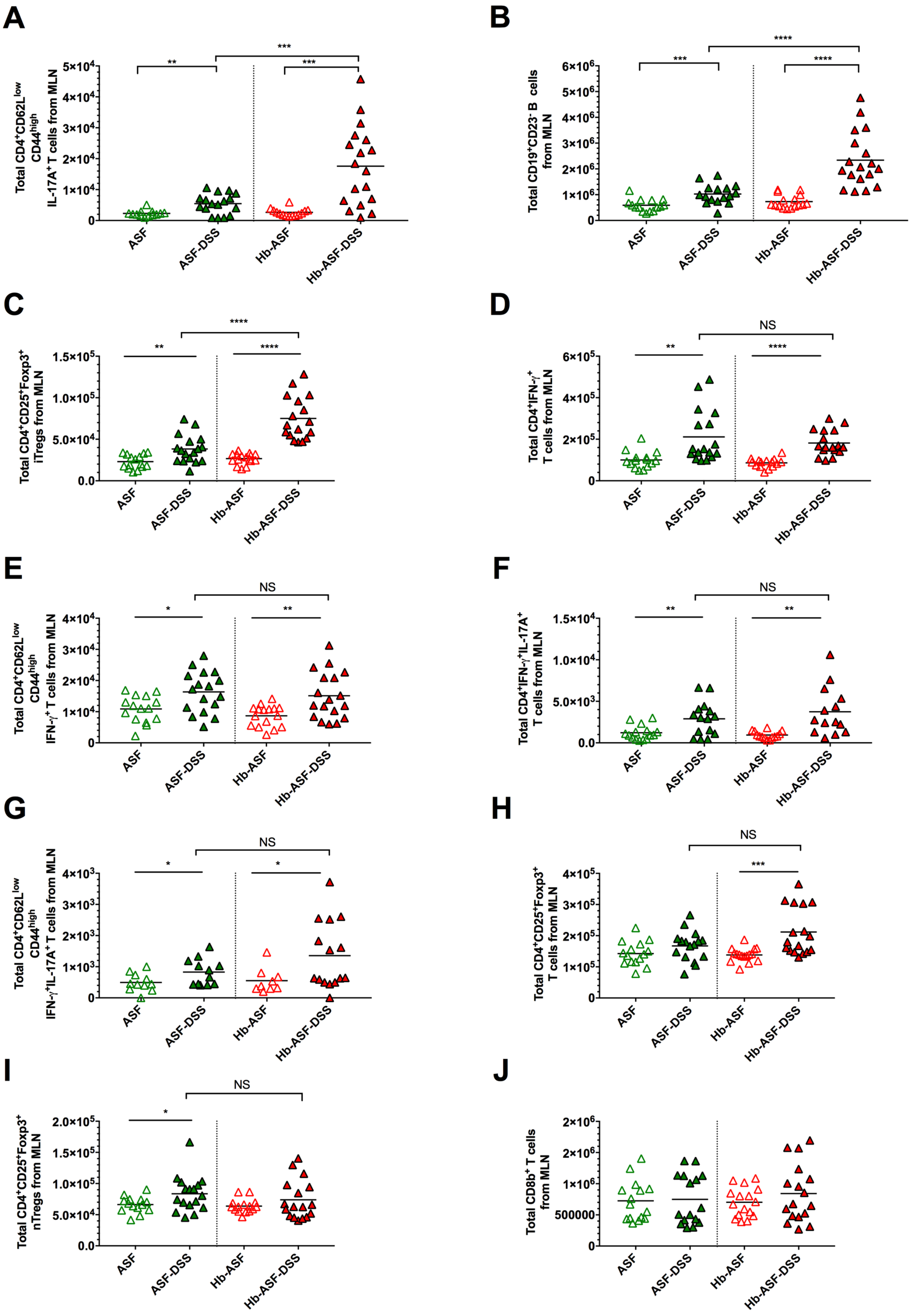
A
Total CD4+CD62Llow CD44high IL-17A+ T cells from MLN
5×10^4
4×10^4
3×10^4
2×10^4
1×10^4
0
**
***
***
ASF
ASF-DSS
Hb-ASF
Hb-ASF-DSS
B
Total CD19+CD23- B cells from MLN
6×10^6
5×10^6
4×10^6
3×10^6
2×10^6
1×10^6
0
***
****
****
ASF
ASF-DSS
Hb-ASF
Hb-ASF-DSS
C
Total CD4+CD25+Foxp3+ iTregs from MLN
1.5×10^5
1.0×10^5
5.0×10^4
0.0
**
****
****
ASF
ASF-DSS
Hb-ASF
Hb-ASF-DSS
D
Total CD4+IFN-γ+ T cells from MLN
6×10^5
4×10^5
2×10^5
0
**
NS
****
ASF
ASF-DSS
Hb-ASF
Hb-ASF-DSS
E
Total CD4+CD62Llow CD44high IFN-γ+ T cells from MLN
4×10^4
3×10^4
2×10^4
1×10^4
0
*
NS
**
ASF
ASF-DSS
Hb-ASF
Hb-ASF-DSS
F
Total CD4+IFN-γ+IL-17A+ T cells from MLN
1.5×10^4
1.0×10^4
5.0×10^3
0.0
**
NS
**
ASF
ASF-DSS
Hb-ASF
Hb-ASF-DSS
G
Total CD4+CD62Llow CD44high IFN-γ+IL-17A+ T cells from MLN
4×10^3
3×10^3
2×10^3
1×10^3
0
*
NS
*
ASF
ASF-DSS
Hb-ASF
Hb-ASF-DSS
H
Total CD4+CD25+Foxp3+ T cells from MLN
4×10^5
3×10^5
2×10^5
1×10^5
0
NS
***
ASF
ASF-DSS
Hb-ASF
Hb-ASF-DSS
I
Total CD4+CD25+Foxp3+ nTregs from MLN
2.0×10^5
1.5×10^5
1.0×10^5
5.0×10^4
0.0
*
NS
ASF
ASF-DSS
Hb-ASF
Hb-ASF-DSS
J
Total CD8b+ T cells from MLN
2×10^6
2×10^6
1×10^6
500000
0
ASF
ASF-DSS
Hb-ASF
Hb-ASF-DSS

Figure S3. Effector memory Th17, active B cells and iTregs, but not nTregs or IFN-γ^+ $CD4^+$ T cells, expanded during pathobiont-induced intestinal inflammation. (**A-F**) Total number of effector memory (EM) $CD44^{high}CD62L^{low}$ IL-17A^+ $CD4^+$ T cells (**A**), active $CD19^+CD23^-$ B cells (**B**), iTreg cells ($CD25^+Foxp3^+Neuropilin^{low}Helios^{low}$ $CD4^+$) (**C**), total number of IFN-γ^+ $CD4^+$ T cells (**D**), EM IFN-γ^+ $CD4^+$ T cells (**E**), total IFN-γ^+ IL-17A^+ $CD4^+$ T cells (**F**), EM IFN-γ^+ IL-17A^+ $CD4^+$ T cells (**G**), total $CD25^+$ $Foxp3^+$ $CD4^+$ Tregs (**H**), nTregs ($CD25^+$ $Foxp3^+$ $Neuropilin^{high}$ $Helios^{high}$ $CD4^+$) (**I**) and $CD8b^+$ T cells (**J**) in mesenteric lymph nodes (MLN; *n* = 9-18 animals per treatment). (**A-J**) Horizontal bars represent treatment means in all graphs. Differences in the absolute immune cell counts were tested using an unpaired parametric T-test using a two-tailed distribution for *P*-value calculations. Asterisks depict the degree of significance for the difference in the total number of immune cells (* $P < 0.05$, ** $P \leq 0.01$, *** $P \leq 0.001$, **** $P \leq 0.0001$ and NS = not significant $P \geq 0.05$). Experiments were performed using male and female C57BL/6 mice at 8-10 wks of age; mice were colonized with *H. bilis* for 3 wks.

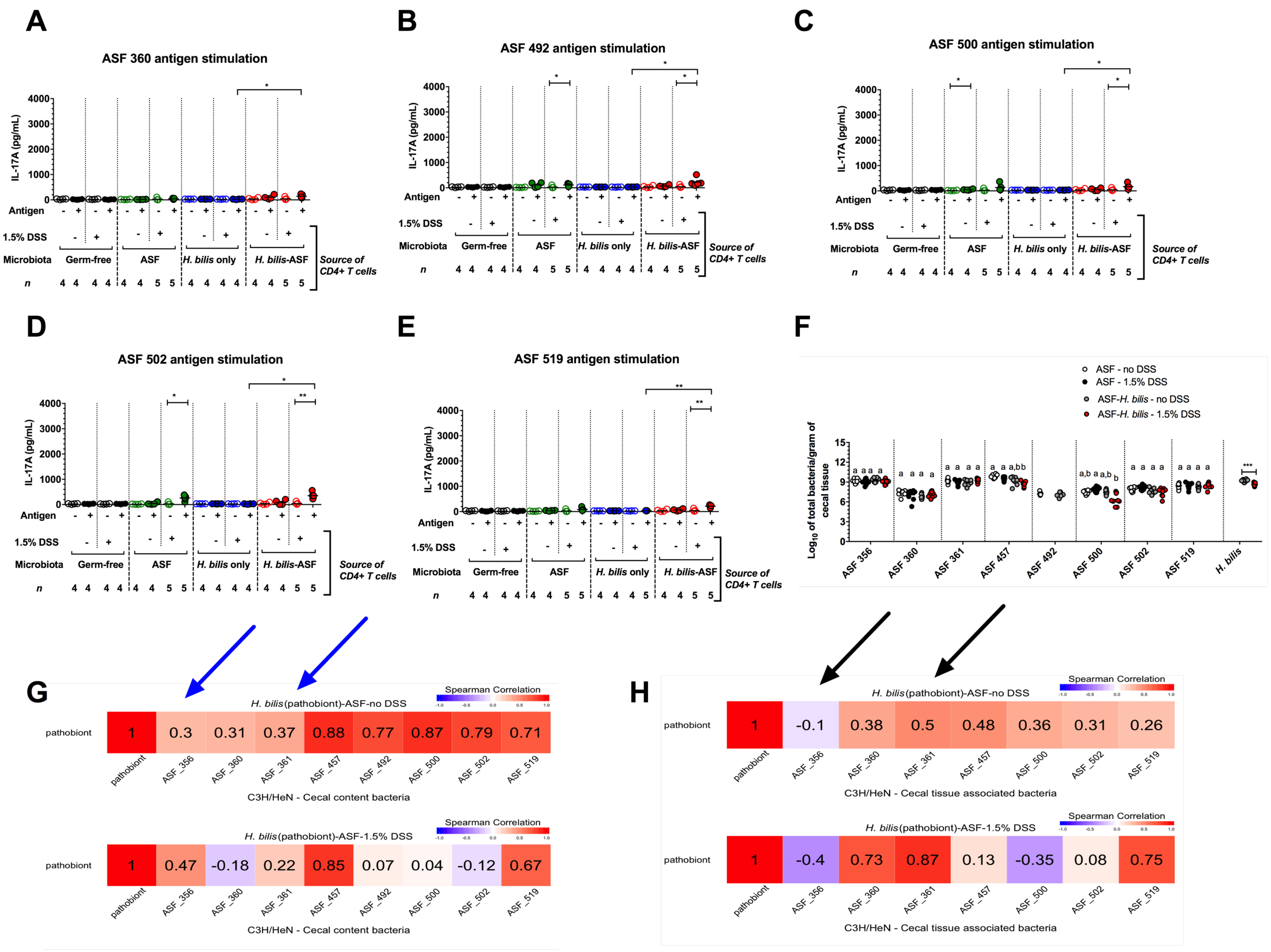
A
ASF 360 antigen stimulation
B
ASF 492 antigen stimulation
C
ASF 500 antigen stimulation
D
ASF 502 antigen stimulation
E
ASF 519 antigen stimulation
IL-17A (pg/mL)
Antigen
1.5% DSS
Microbiota
Germ-free
ASF
H. bilis only
H. bilis-ASF
Source of CD4+ T cells
n
F
ASF - no DSS
ASF - 1.5% DSS
ASF-H. bilis - no DSS
ASF-H. bilis - 1.5% DSS
Log10 of total bacteria/gram of cecal tissue
ASF 356
ASF 360
ASF 361
ASF 457
ASF 492
ASF 500
ASF 502
ASF 519
H. bilis
G
H. bilis (pathobiont)-ASF-no DSS
Spearman Correlation
pathobiont
1
0.3
0.31
0.37
0.88
0.77
0.87
0.79
0.71
pathobiont
ASF_356
ASF_360
ASF_361
ASF_457
ASF_492
ASF_500
ASF_502
ASF_519
C3H/HeN - Cecal content bacteria
H. bilis (pathobiont)-ASF-1.5% DSS
Spearman Correlation
pathobiont
1
0.47
-0.18
0.22
0.85
0.07
0.04
-0.12
0.67
C3H/HeN - Cecal content bacteria
H
H. bilis (pathobiont)-ASF-no DSS
Spearman Correlation
pathobiont
1
-0.1
0.38
0.5
0.48
0.36
0.31
0.26
C3H/HeN - Cecal tissue associated bacteria
H. bilis (pathobiont)-ASF-1.5% DSS
Spearman Correlation
pathobiont
1
-0.4
0.73
0.87
0.13
-0.35
0.08
0.75
C3H/HeN - Cecal tissue associated bacteria

Figure S4. Pathobiont-mediated intestinal inflammation triggered Th17 immunoreactivity against specific ASF members in the absence of increased abundance or co-abundance of the targeted taxa in cecal contents or tissue. (**A-E**) IL-17A secretion by $CD4^+$ T cells isolated from the mesenteric lymph nodes of germ-free (GF), ASF-bearing, *H. bilis* mono-associated and *H. bilis* colonized ASF-bearing mice treated with 1.5% DSS or left untreated. $CD4^+$ T cells were either left unstimulated (NS) or stimulated with whole-cell sonicate antigens from either ASF 360 (*Lactobacillus intestinalis*) (**A**), ASF 492 (*E. plexicaudatum*) (**B**), ASF 500 (*Pseudoflavonifractor sp.*) (**C**), ASF 502 (*Clostridium sp.*) (**D**) or ASF 519 (*Parabacteroides goldsteinii*) (**E**) for 72 hrs (n = 4-5 pools of 2-3 animals per pool per treatment). (**F**) Cecal tissue-associated bacterial abundances in *H. bilis* colonized ASF-bearing mice treated with 1.5% DSS or left untreated (n = 6-9 animals per treatment). (**G-H**) Spearman's correlation coefficient between *H. bilis* and each individual ASF bacterial abundance in *H. bilis* colonized ASF-bearing mice treated with 1.5% DSS or left untreated (n = 8-10 animals per treatment). Arrows highlight the absence of strong correlations between the immune-dominant species ASF 356 (*Clostridium sp.)* or ASF 361 (*Lactobacillus murinus*) with the pathobiont *H. bilis* during disease (DSS) versus no disease (no DSS) in either cecal contents (**G** – blue arrows) or tissues (**H** – black arrows). (**A-E**) Asterisks depict the degree of significance determined by a non-parametric unpaired Mann-Whitney test using a two-tailed distribution for P-value calculations (* $P < 0.05$, ** $P \leq 0.01$, *** $P \leq 0.001$ and **** $P \leq 0.0001$ and NS = not significant $P \geq 0.05$). (F) Differing superscript letters indicate significant differences across treatments as per a non-parametric Kruskal-Wallis one-way ANOVA, followed by a post-hoc test (Dunn's test, $P < 0.05$). ASF bacterial abundances were measured using species-specific qPCR assays. All experiments were performed using male and female C3H/HeN mice.

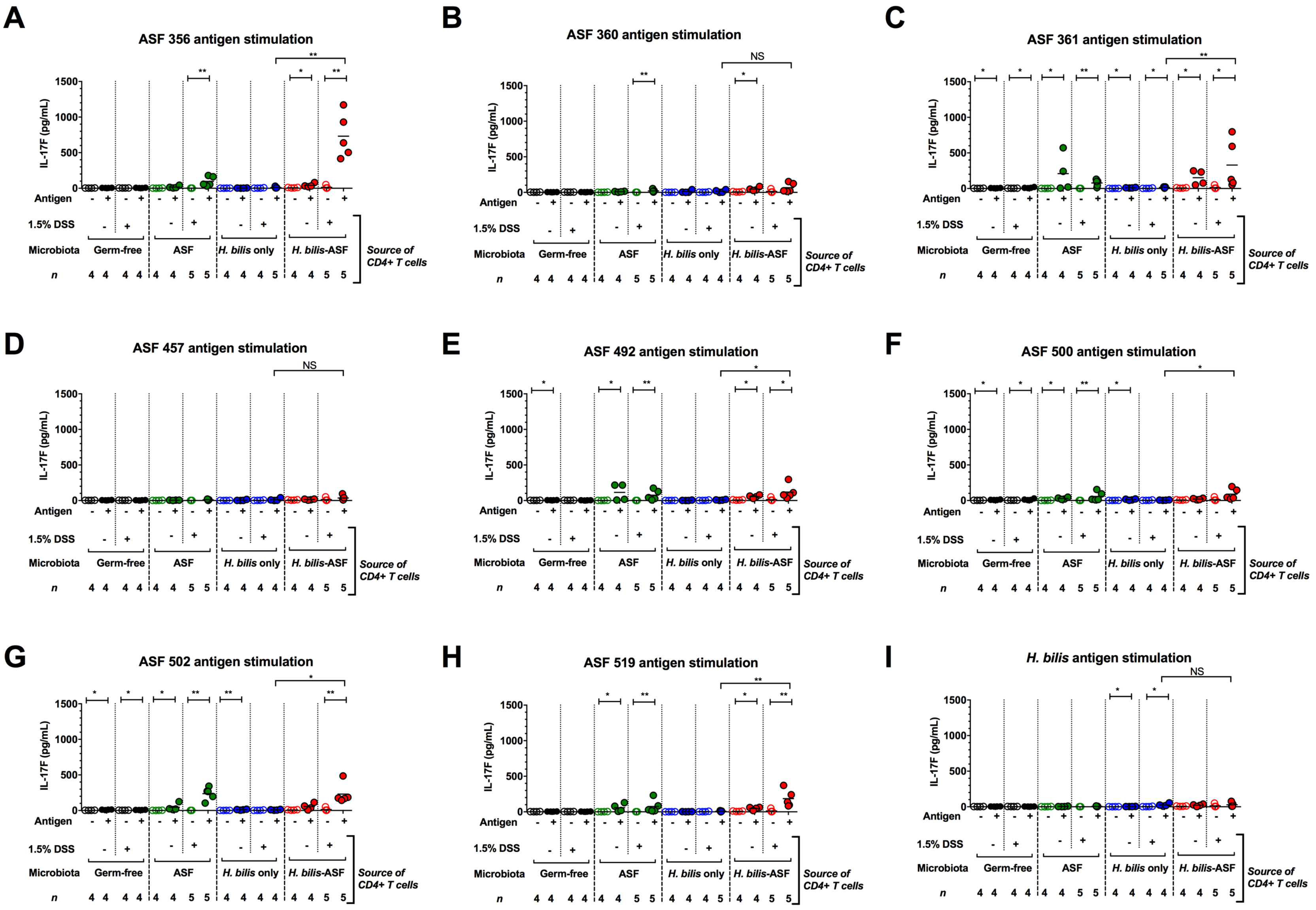
A
ASF 356 antigen stimulation
B
ASF 360 antigen stimulation
C
ASF 361 antigen stimulation
D
ASF 457 antigen stimulation
E
ASF 492 antigen stimulation
F
ASF 500 antigen stimulation
G
ASF 502 antigen stimulation
H
ASF 519 antigen stimulation
I
H. bilis antigen stimulation
IL-17F (pg/mL)
1500
1000
500
0
NS
Antigen
1.5% DSS
Microbiota
Germ-free
ASF
H. bilis only
H. bilis-ASF
n
Source of CD4+ T cells

Figure S5. $CD4^+$ T cells from *H. bilis* colonized ASF-bearing mice treated with 1.5% DSS secreted IL-17F primarily in response to ASF 356 (*Clostridium sp.*) and ASF 361 (*L. murinus*). (**A-I**) IL-17F secretion by $CD4^+$ T cells isolated from the mesenteric lymph nodes of germ-free (GF), ASF-bearing, *H. bilis* mono-associated and *H. bilis* colonized ASF-bearing mice treated with 1.5% DSS or left untreated. $CD4^+$ T cells were either left unstimulated (NS) or stimulated with whole-cell sonicate antigens from either ASF 356 (*Clostridium sp.*) (**A**), ASF 360 (*Lactobacillus intestinalis*) (**B**), ASF 361 (*Lactobacillus murinus*) (**C**), ASF 457 (*Mucispirillum schaedleri*) (**D**), ASF 492 (*Eubacterium plexicaudatum*) (**E**), ASF 500 (*Pseudoflavonifractor sp.*) (**F**), ASF 502 (*Clostridium sp.*) (**G**), ASF 519 (*Parabacteroides goldsteinii*) (**H**) or the pathobiont *H. bilis* (**I**) for 72 hrs (n = 4-5 pools of 2-3 animals per pool per treatment). Asterisks depict the degree of significance for differences as determined by a non-parametric unpaired Mann-Whitney test using a two-tailed distribution for P-value calculations (* $P < 0.05$, ** $P \leq 0.01$, *** $P \leq 0.001$, **** $P \leq 0.0001$ and NS = not significant $P \geq 0.05$). Experiments were performed using male and female C3H/HeN mice at 8-10 wks of age; mice were colonized with *H. bilis* for 3 wks.

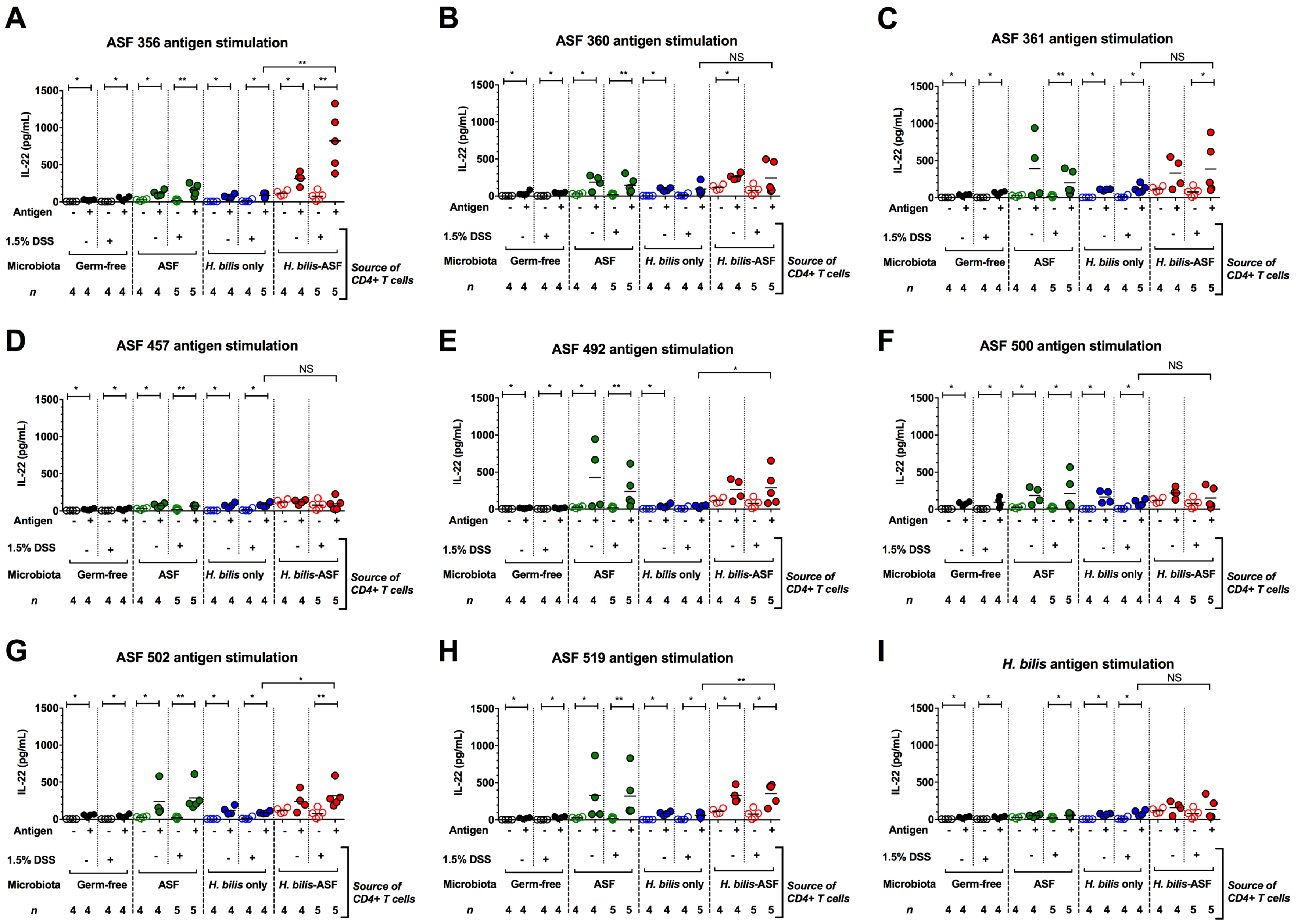
A
ASF 356 antigen stimulation
B
ASF 360 antigen stimulation
C
ASF 361 antigen stimulation
D
ASF 457 antigen stimulation
E
ASF 492 antigen stimulation
F
ASF 500 antigen stimulation
G
ASF 502 antigen stimulation
H
ASF 519 antigen stimulation
I
H. bilis antigen stimulation
IL-22 (pg/mL)
Antigen
1.5% DSS
Microbiota
Germ-free
ASF
H. bilis only
H. bilis-ASF
n
Source of CD4+ T cells

Figure S6. $CD4^+$ T cells from *H. bilis* colonized ASF-bearing mice treated with 1.5% DSS secreted IL-22 primarily in response to ASF 356 (*Clostridium sp.*). (**A-I**) IL-22 secretion by $CD4^+$ T cells isolated from the mesenteric lymph nodes of germ-free (GF), ASF-bearing, *H. bilis* mono-associated and *H. bilis* colonized ASF-bearing mice treated with 1.5% DSS or left untreated. $CD4^+$ T cells were either left unstimulated (NS) or stimulated with whole-cell sonicate antigens from either ASF 356 (*Clostridium sp.*) (**A**), ASF 360 (*Lactobacillus intestinalis*) (**B**), ASF 361 (*Lactobacillus murinus*) (**C**), ASF 457 (*Mucispirillum schaedleri*) (**D**), ASF 492 (*Eubacterium plexicaudatum*) (**E**), ASF 500 (*Pseudoflavonifractor sp.*) (**F**), ASF 502 (*Clostridium sp.*) (**G**), ASF 519 (*Parabacteroides goldsteinii*) (**H**) or the pathobiont *H. bilis* (**I**) for 72 hrs (n = 4-5 pools of 2-3 animals per pool per treatment). Asterisks depict the degree of significance for differences as determined by a non-parametric unpaired Mann-Whitney test using a two-tailed distribution for P-value calculations (* $P < 0.05$, ** $P \leq 0.01$, *** $P \leq 0.001$, **** $P \leq 0.0001$ and NS = not significant $P \geq 0.05$). Experiments were performed using male and female C3H/HeN mice at 8-10 wks of age; mice were colonized with *H. bilis* for 3 wks.

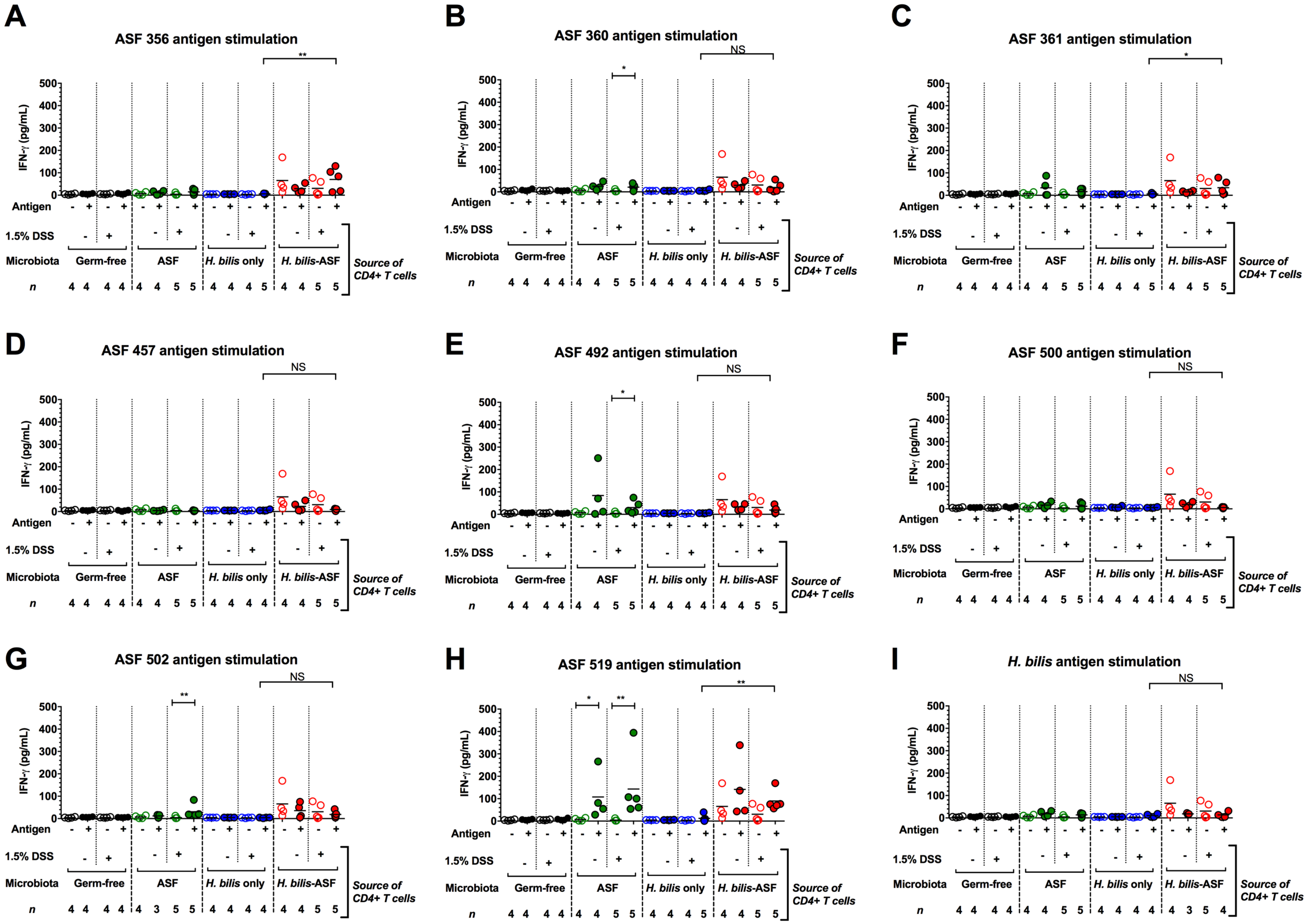
A
ASF 356 antigen stimulation
B
ASF 360 antigen stimulation
C
ASF 361 antigen stimulation
D
ASF 457 antigen stimulation
E
ASF 492 antigen stimulation
F
ASF 500 antigen stimulation
G
ASF 502 antigen stimulation
H
ASF 519 antigen stimulation
I
H. bilis antigen stimulation
IFN-γ (pg/mL)
Antigen
1.5% DSS
Microbiota
Germ-free
ASF
H. bilis only
H. bilis-ASF
n
Source of CD4+ T cells
NS

Figure S7. Antigen-specific IFN-γ production by $CD4^+$ T cells from ASF-bearing mice was not a hallmark of pathobiont-induced intestinal inflammation. (**A-I**) IFN-γ secretion by $CD4^+$ T cells isolated from the mesenteric lymph nodes of germ-free (GF), ASF-bearing, *H. bilis* mono-associated and *H. bilis* colonized ASF-bearing mice treated with 1.5% DSS or left untreated. $CD4^+$ T cells were either left unstimulated (NS) or stimulated with whole-cell sonicate antigens from either ASF 356 (*Clostridium sp.*) (**A**), ASF 360 (*Lactobacillus intestinalis*) (**B**), ASF 361 (*Lactobacillus murinus*) (**C**), ASF 457 (*Mucispirillum schaedleri*) (**D**), ASF 492 (*Eubacterium plexicaudatum*) (**E**), ASF 500 (*Pseudoflavonifractor sp.*) (**F**), ASF 502 (*Clostridium sp.*) (**G**), ASF 519 (*Parabacteroides goldsteinii*) (**H**) or the pathobiont *H. bilis* (**I**) for 72 hrs (n = 4-5 pools of 2-3 animals per pool per treatment). Asterisks depict the degree of significance for differences as determined by a non-parametric unpaired Mann-Whitney test using a two-tailed distribution for P-value calculations (* $P < 0.05$, ** $P \leq 0.01$, *** $P \leq 0.001$, **** $P \leq 0.0001$ and NS = not significant $P \geq 0.05$). Experiments were performed using male and female C3H/HeN mice at 8-10 wks of age; mice were colonized with *H. bilis* for 3 wks.

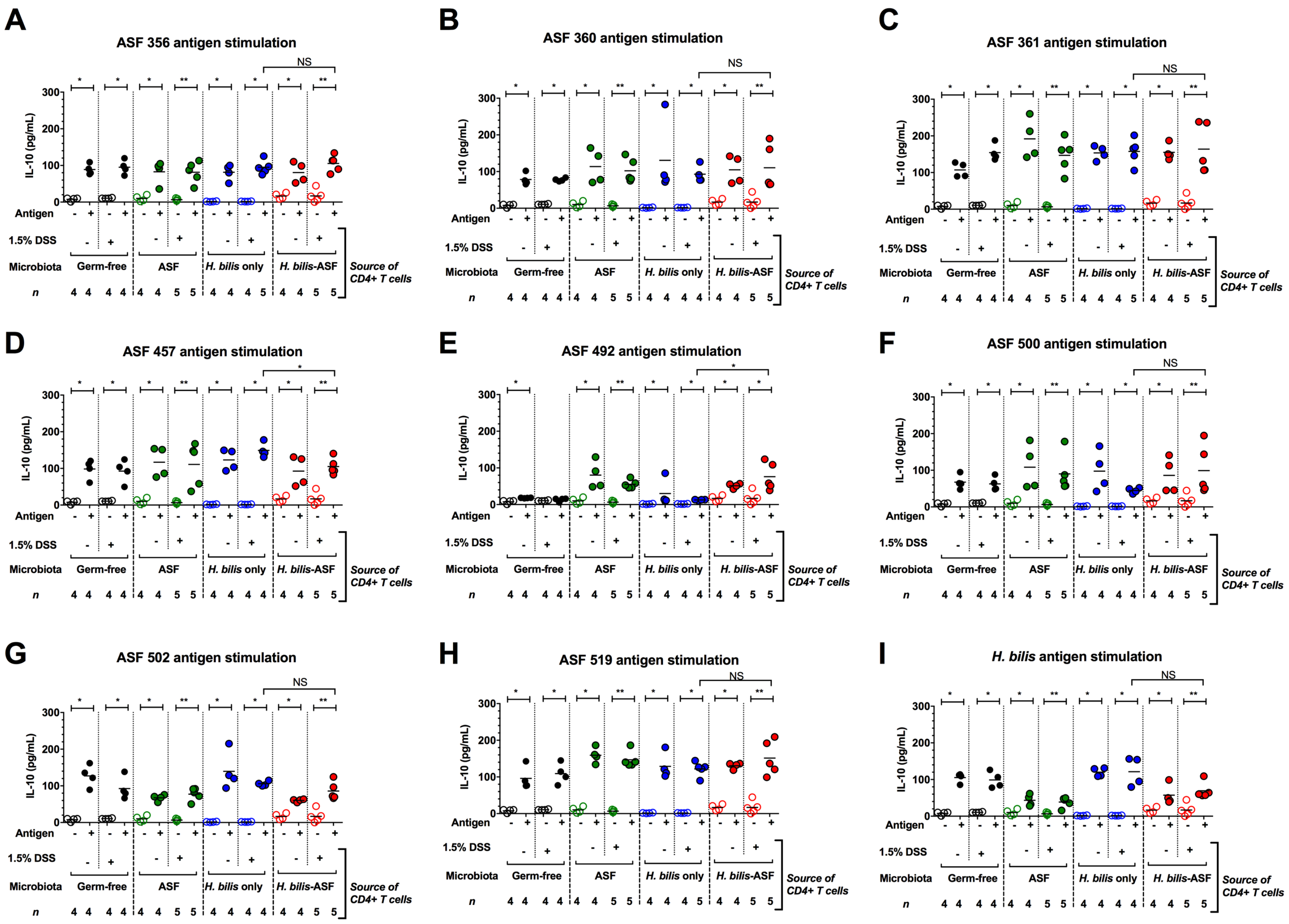
A
ASF 356 antigen stimulation
B
ASF 360 antigen stimulation
C
ASF 361 antigen stimulation
D
ASF 457 antigen stimulation
E
ASF 492 antigen stimulation
F
ASF 500 antigen stimulation
G
ASF 502 antigen stimulation
H
ASF 519 antigen stimulation
I
H. bilis antigen stimulation
IL-10 (pg/mL)
NS
Antigen
1.5% DSS
Microbiota
Germ-free
ASF
H. bilis only
H. bilis-ASF
n
Source of CD4+ T cells

Figure S8. Antigen-specific IL-10 production by $CD4^+$ T cells from ASF-bearing mice was not a hallmark of pathobiont-induced intestinal inflammation. (**A-I**) IL-10 secretion by $CD4^+$ T cells isolated from the mesenteric lymph nodes of germ-free (GF), ASF-bearing, *H. bilis* mono-associated and *H. bilis* colonized ASF-bearing mice treated with 1.5% DSS or left untreated. $CD4^+$ T cells were either left unstimulated (NS) or stimulated with whole-cell sonicate antigens from either ASF 356 (*Clostridium sp.*) (**A**), ASF 360 (*Lactobacillus intestinalis*) (**B**), ASF 361 (*Lactobacillus murinus*) (**C**), ASF 457 (*Mucispirillum schaedleri*) (**D**), ASF 492 (*Eubacterium plexicaudatum*) (**E**), ASF 500 (*Pseudoflavonifractor sp.*) (**F**), ASF 502 (*Clostridium sp.*) (**G**), ASF 519 (*Parabacteroides goldsteinii*) (**H**) or the pathobiont *H. bilis* (**I**) for 72 hrs (n = 4-5 pools of 2-3 animals per pool per treatment). Asterisks depict the degree of significance for differences as determined by a non-parametric unpaired Mann-Whitney test using a two-tailed distribution for P-value calculations (* $P < 0.05$, ** $P \leq 0.01$, *** $P \leq 0.001$, **** $P \leq 0.0001$ and NS = not significant $P \geq 0.05$). Experiments were performed using male and female C3H/HeN mice at 8-10 wks of age; mice were colonized with *H. bilis* for 3 wks.

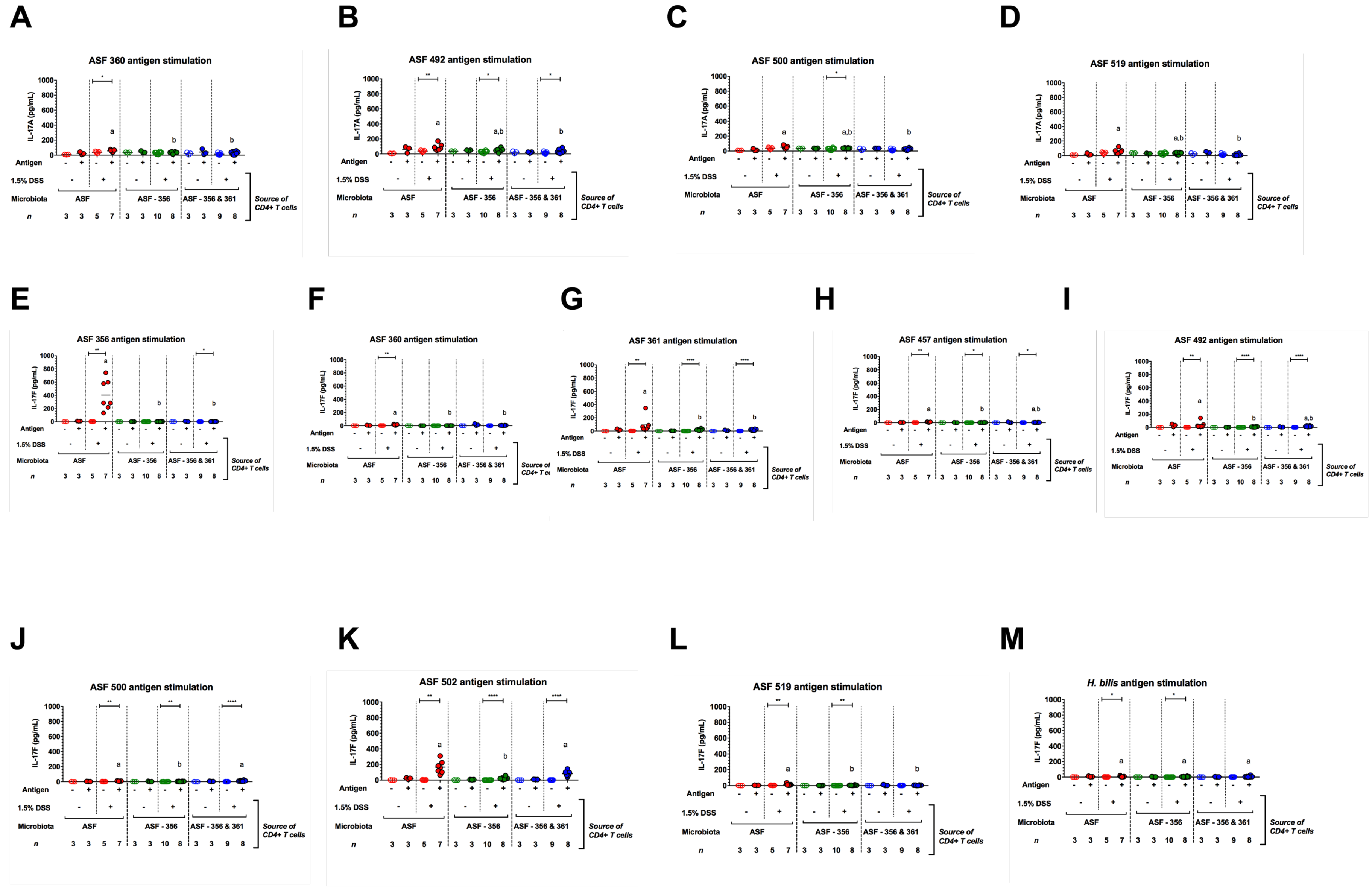
A
ASF 360 antigen stimulation
IL-17A (pg/mL)
B
ASF 492 antigen stimulation
IL-17A (pg/mL)
C
ASF 500 antigen stimulation
IL-17A (pg/mL)
D
ASF 519 antigen stimulation
IL-17A (pg/mL)
E
ASF 356 antigen stimulation
IL-17F (pg/mL)
F
ASF 360 antigen stimulation
IL-17F (pg/mL)
G
ASF 361 antigen stimulation
IL-17F (pg/mL)
H
ASF 457 antigen stimulation
IL-17F (pg/mL)
I
ASF 492 antigen stimulation
IL-17F (pg/mL)
J
ASF 500 antigen stimulation
IL-17F (pg/mL)
K
ASF 502 antigen stimulation
IL-17F (pg/mL)
L
ASF 519 antigen stimulation
IL-17F (pg/mL)
M
H. bilis antigen stimulation
IL-17F (pg/mL)
Antigen
1.5% DSS
Microbiota
ASF
ASF - 356
ASF - 356 & 361
n
Source of CD4+ T cells

Figure S9. Removal of immune-dominant taxa from the resident microbiota does not alter the severity of pathobiont-mediated intestinal inflammation or eliminate Th17 immunoreactivity against the remaining gut symbionts. IL-17A (**A-D**) and IL-17F (**E-M**) secretion from $CD4^+$ T cells isolated from the mesenteric lymph nodes of mice in all treatments except for animals colonized with the ASF minus 457. $CD4^+$ T cells were either left unstimulated (NS) or stimulated with individual whole-cell sonicate ASF antigens for 72 hrs (n = 2-24 pools of 2-3 animals per pool per treatment). (**A-M**) Asterisks depict the degree of significance for differences as determined by a non-parametric unpaired Mann-Whitney test using a two-tailed distribution for P-value calculations (* $P < 0.05$, ** $P \leq 0.01$, *** $P \leq 0.001$, **** $P \leq 0.0001$ and NS = not significant $P \geq 0.05$). Differing superscript letters indicate significant differences across treatments as per a non-parametric Kruskal-Wallis one-way ANOVA, followed by a post-hoc test (Dunn's test, $P < 0.05$). Experiments were initiated when male and female C3H/HeN mice were 4-5 wks of age.

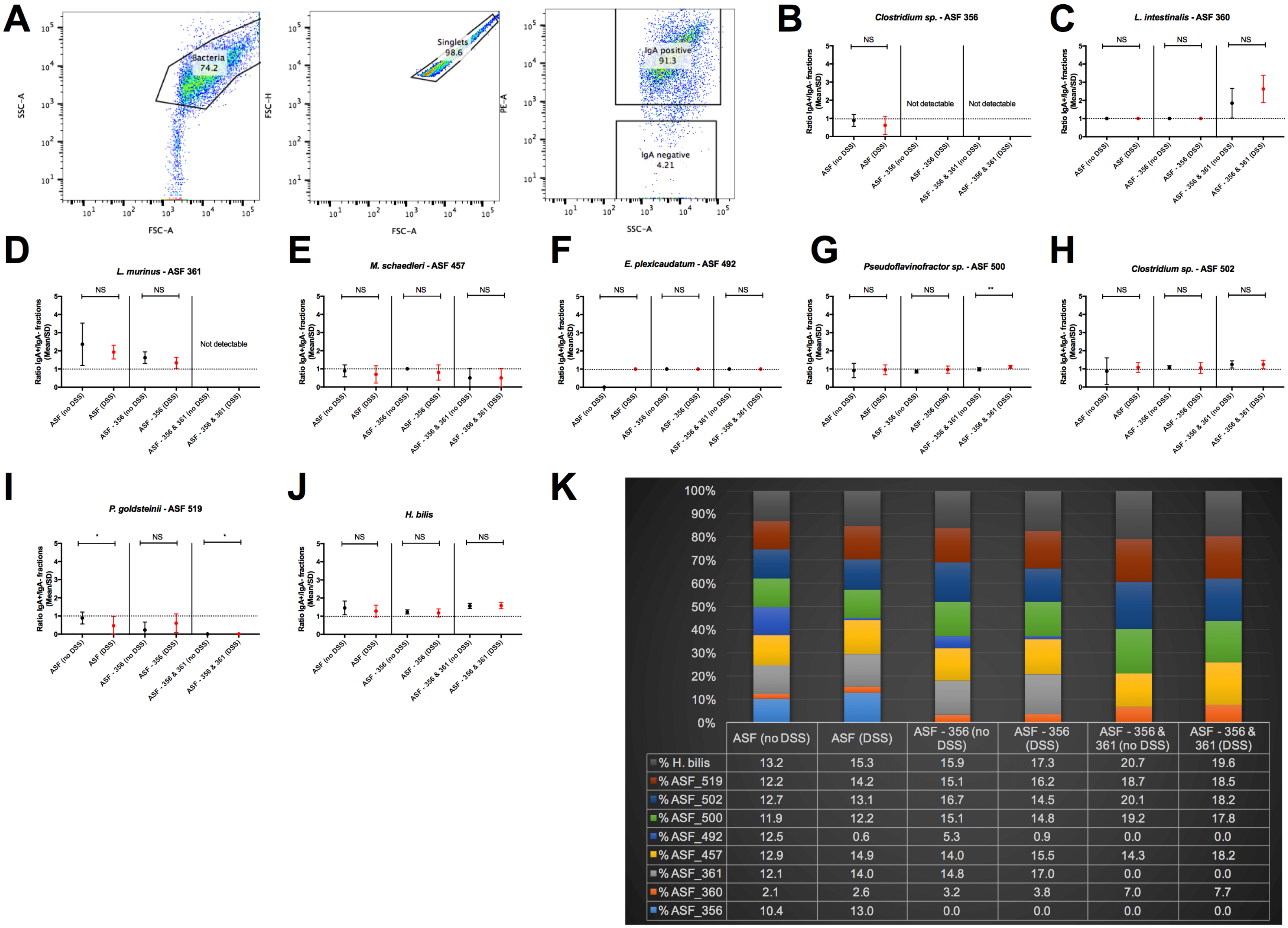

	ASF (no DSS)	ASF (DSS)	ASF - 356 (no DSS)	ASF - 356 (DSS)	ASF - 356 & 361 (no DSS)	ASF - 356 & 361 (DSS)
% H. bilis	13.2	15.3	15.9	17.3	20.7	19.6
% ASF_519	12.2	14.2	15.1	16.2	18.7	18.5
% ASF_502	12.7	13.1	16.7	14.5	20.1	18.2
% ASF_500	11.9	12.2	15.1	14.8	19.2	17.8
% ASF_492	12.5	0.6	5.3	0.9	0.0	0.0
% ASF_457	12.9	14.9	14.0	15.5	14.3	18.2
% ASF_361	12.1	14.0	14.8	17.0	0.0	0.0
% ASF_360	2.1	2.6	3.2	3.8	7.0	7.7
% ASF_356	10.4	13.0	0.0	0.0	0.0	0.0

Figure S10. Absence of enhanced IgA coating of intestinal bacteria during pathobiont-mediated intestinal inflammation. **(A)** FACS gating strategy to sort the bacterial IgA+ fraction from cecal contents for subsequent quantification of the ASF and *H. bilis* relative abundances by qPCR. FACS was performed following pre-enrichment of the IgA+ fraction using magnetic cell sorting. **(B-K)** Intestinal inflammation triggered by *H. bilis* and DSS treatment did not significantly affect the ratio of bacteria detected in the IgA+/- fractions (n = 5-13 individual cecal samples per treatment). A ratio of 1 between IgA+ and – fractions indicates equal bacterial relative abundances (represented by the horizontal dotted line in Fig. S10B-J). (**B-J**) Asterisks depict the degree of significance for differences as determined by a parametric unpaired T-test using a two-tailed distribution for P-value calculations (* $P < 0.05$, ** $P \leq 0.01$, *** $P \leq 0.001$, **** $P \leq 0.0001$ and NS = not significant $P \geq 0.05$). Experiments were initiated when male and female C3H/HeN mice were 4-5 wks of age. Experimental design is described in Fig. 5A.

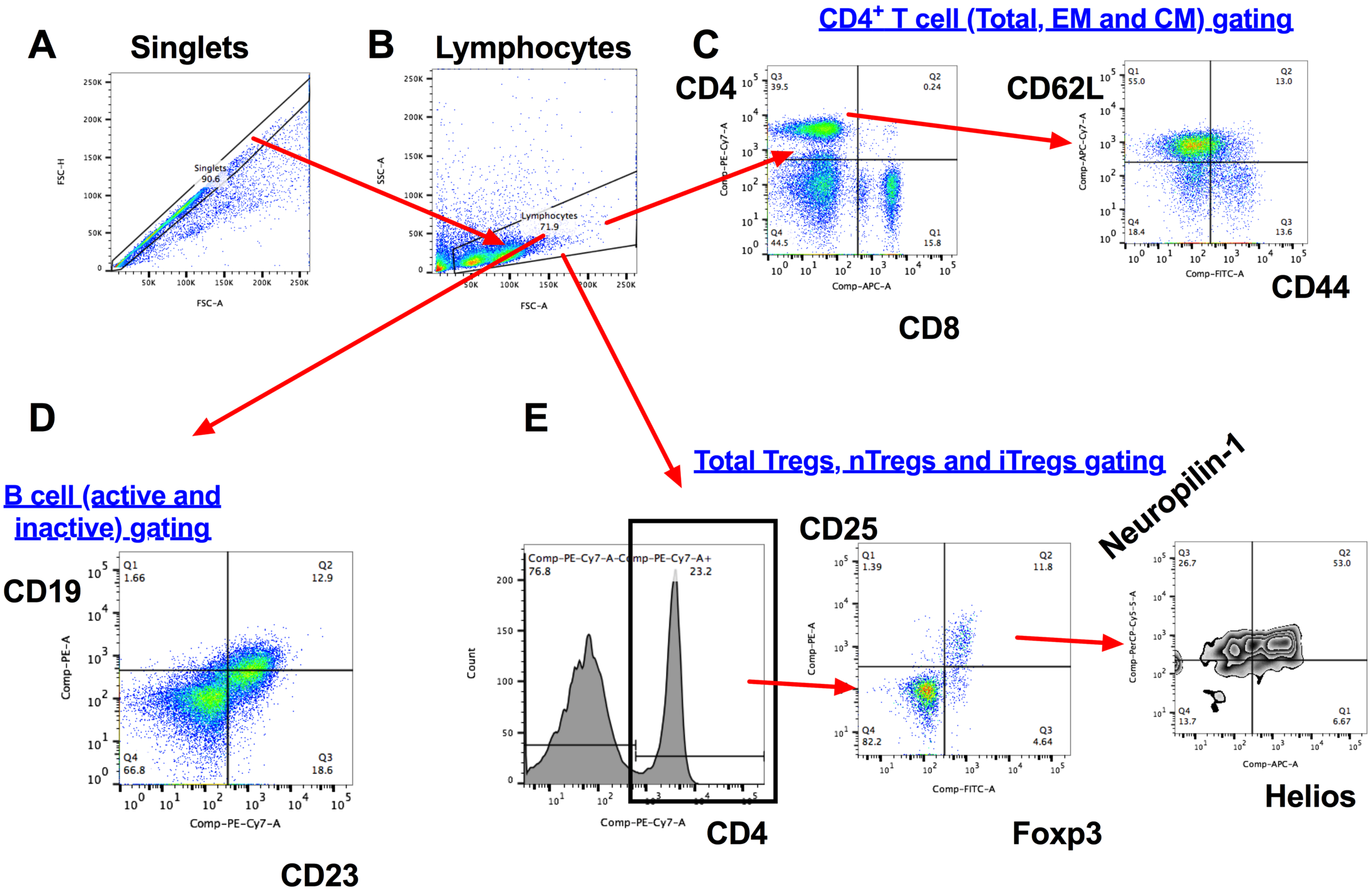

A
Singlets
Singlets
90.6
FSC-H
FSC-A
B
Lymphocytes
Lymphocytes
71.9
SSC-A
FSC-A
C
CD4⁺ T cell (Total, EM and CM) gating
CD4
Q3
39.5
Q2
0.24
Q4
44.5
Q1
15.8
Comp-PE-Cy7-A
Comp-APC-A
CD8
CD62L
Q1
55.0
Q2
13.0
Q4
18.4
Q3
13.6
Comp-APC-Cy7-A
Comp-FITC-A
CD44
D
B cell (active and inactive) gating
CD19
Q1
1.66
Q2
12.9
Q4
66.8
Q3
18.6
Comp-PE-A
Comp-PE-Cy7-A
CD23
E
Total Tregs, nTregs and iTregs gating
Comp-PE-Cy7-A-
76.8
Comp-PE-Cy7-A+
23.2
Count
Comp-PE-Cy7-A
CD4
CD25
Q1
1.39
Q2
11.8
Q4
82.2
Q3
4.64
Comp-PE-A
Comp-FITC-A
Foxp3
Neuropilin-1
Q3
26.7
Q2
53.0
Q4
13.7
Q1
6.67
Comp-PerCP-Cy5-5-A
Comp-APC-A
Helios

Figure S11. Flow cytometry gating strategy for quantification of lymphocytes in the mesenteric lymph nodes during pathobiont-elicited intestinal inflammation. **(A-B)** Gating on singlets and lymphocytes, respectively. **(C)** Gating on $CD4^{+}$ or $CD8b^{+}$ T cells. **(D)** Gating on effector or central memory (EM-$CD62L^{low}$ $CD44^{high}$ or CM-$CD62L^{high}$ $CD44^{high}$) $CD4^{+}$ T cells. **(D)** Gating on activated ($CD19^{high}$ $CD23^{low}$) B cell populations. **(E)** Gating on total Tregs ($CD25^{+}$ $Foxp3^{+}$ $CD4^{+}$), nTregs ($CD25^{+}$ $Foxp3^{+}$ Neuropilin-1^{high} $Helios^{high}$ $CD4^{+}$) and iTregs ($CD25^{+}$ $Foxp3^{+}$ Neuropilin-1^{low} $Helios^{low}$ $CD4^{+}$).

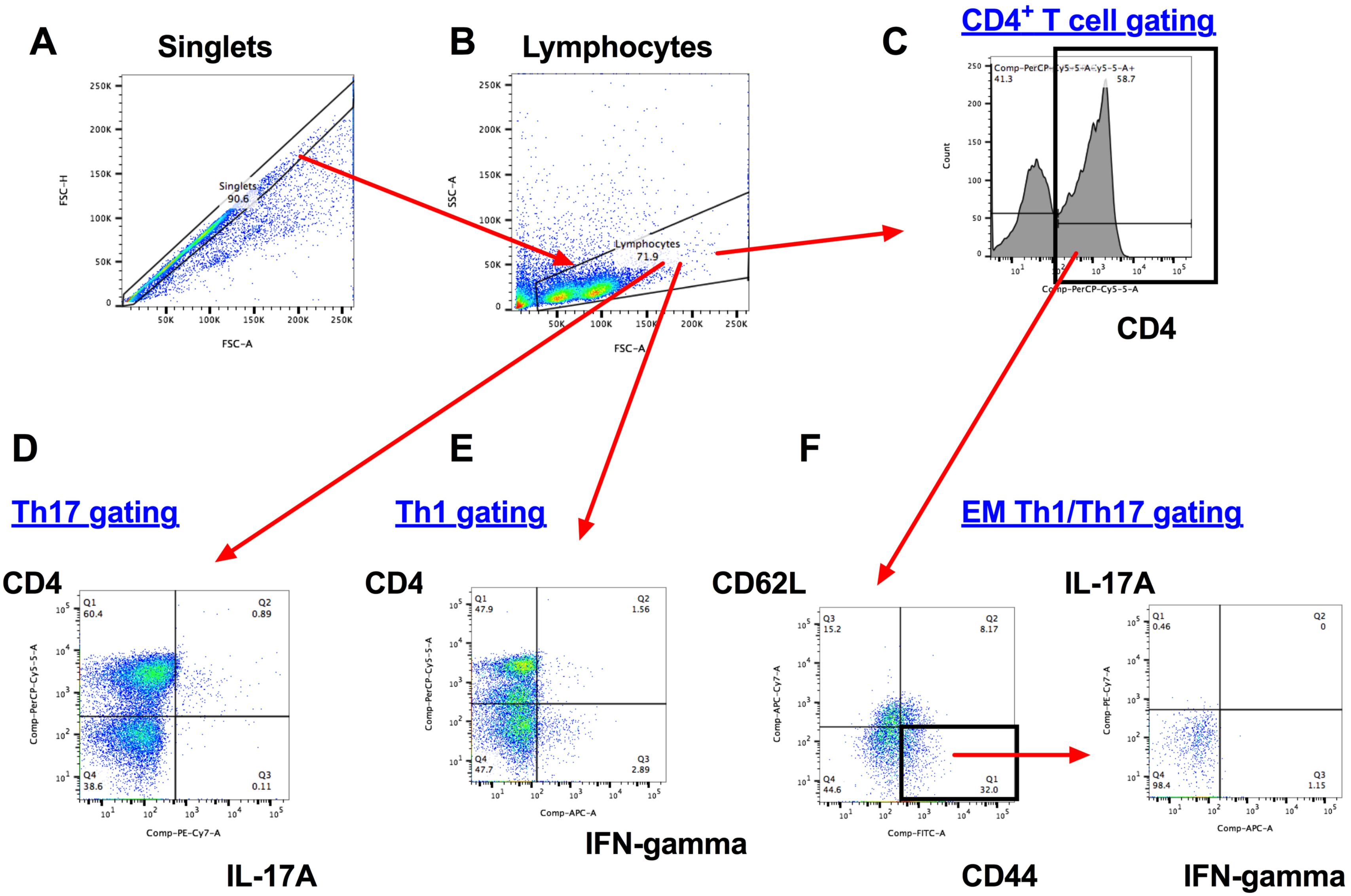
A
Singlets
Singlets
90.6
FSC-H
FSC-A
B
Lymphocytes
Lymphocytes
71.9
SSC-A
FSC-A
C
CD4+ T cell gating
41.3
58.7
Count
Comp-PerCP-Cy5-5-A
CD4
D
Th17 gating
CD4
Q1
60.4
Q2
0.89
Q3
0.11
Q4
38.6
Comp-PerCP-Cy5-5-A
Comp-PE-Cy7-A
IL-17A
E
Th1 gating
CD4
Q1
47.9
Q2
1.56
Q3
2.89
Q4
47.7
Comp-PerCP-Cy5-5-A
Comp-APC-A
IFN-gamma
F
EM Th1/Th17 gating
CD62L
Q3
15.2
Q2
8.17
Q1
32.0
Q4
44.6
Comp-APC-Cy7-A
Comp-FITC-A
CD44
IL-17A
Q1
0.46
Q2
0
Q3
1.15
Q4
98.4
Comp-PE-Cy7-A
Comp-APC-A
IFN-gamma

Figure S12. Flow cytometry gating strategy for quantification of Th1 and Th17 cells in the mesenteric lymph nodes during pathobiont-elicited intestinal inflammation. **(A-B)** Gating on singlets and lymphocytes, respectively. **(C-E)** Gating on total $CD4^+$ T cells, Th17 (IL-$17A^+$ $CD4^+$) and Th1 cells (IFN-γ^+ $CD4^+$). **(F)** Gating on effector memory Th17 ($CD62L^{low}$ $CD44^{high}$ IL-$17A^+$ $CD4^+$) and Th1 cells ($CD62L^{low}$ $CD44^{high}$ IFN-γ^+ $CD4^+$) .

Supplementary References

1 Gomes-Neto, J. C. *et al.* A real-time PCR assay for accurate quantification of the individual members of the Altered Schaedler Flora microbiota in gnotobiotic mice. *J Microbiol Methods* **135**, 52-62, (2017).

2 Martinez, I. *et al.* Diet-induced metabolic improvements in a hamster model of hypercholesterolemia are strongly linked to alterations of the gut microbiota. *Appl Environ Microbiol* **75**, 4175-4184, (2009).

3 Riley, L. K., Franklin, C. L., Hook, R. R., Jr. & Besch-Williford, C. Identification of murine helicobacters by PCR and restriction enzyme analyses. *J Clin Microbiol* **34**, 942-946, (1996).

4 Ramer-Tait, A. E., Petersen, C. A. & Jones, D. E. IL-2 limits IL-12 enhanced lymphocyte proliferation during *Leishmania amazonensis* infection. *Cell Immunol* **270**, 32-39, (2011).

5 Jergens, A. E. *et al. Helicobacter bilis* triggers persistent immune reactivity to antigens derived from the commensal bacteria in gnotobiotic C3H/HeN mice. *Gut* **56**, 934-940, (2007).

6 Ramer, A. E., Vanloubbeeck, Y. F. & Jones, D. E. Antigen-responsive CD4+ T cells from C3H mice chronically infected with *Leishmania amazonensis* are impaired in the transition to an effector phenotype. *Infect Immun* **74**, 1547-1554, (2006).

7 Palm, N. W. *et al.* Immunoglobulin A coating identifies colitogenic bacteria in inflammatory bowel disease. *Cell* **158**, 1000-1010, (2014).
